# Cerebrospinal fluid protein and glucose levels in neonates with a systemic inflammatory response without meningitis

**DOI:** 10.1186/s12987-018-0095-4

**Published:** 2018-03-14

**Authors:** Mona Noureldein, Roxana Mardare, Jack Pickard, Hoi Lun Shing, Michael Eisenhut

**Affiliations:** Luton & Dunstable University Hospital NHS Foundation Trust, Lewsey Road, Luton, LU4ODZ UK

## Abstract

**Background:**

It has been estimated that paediatric meningitis without elevated CSF white cell count (pleocytosis) accounts for 0.5–12% of all cases of bacterial meningitis. CSF protein and glucose measurements are therefore essential in management but may be neglected in clinical practice. In order to improve recognition of bacterial meningitis in neonates and to enable adequate management and audit, we investigated whether a systemic inflammatory response in the absence of meningitis is associated with elevated CSF protein and reduced CSF glucose levels. A further aim was to determine whether abnormal levels of these parameters were associated with increased incidence of neurological damage.

**Methods:**

As part of an audit into management of abnormal CSF findings in neonates, we conducted a retrospective analysis of neonates without meningitis as evident from normal CSF white blood cell counts and negative CSF culture. We compared data from neonates with fever (temperature > 38.0 °C) and/or elevated C-reactive protein (CRP) levels (> 5 mg/l) (possible sepsis) with data from neonates without fever or CRP elevation.

**Results:**

We analysed results from a total of 244 neonates. CSF protein levels were 0.89 g/l (SD 0.37) in neonates without fever or elevated CRP (n = 26) and not significantly different from neonates with possible sepsis (n = 218) with 0.92 g/l (SD 0.40). CSF glucose levels in infants with possible sepsis were 2.71 (SD 0.83) mmol/l and not significantly different from infants without sepsis with 2.55 mmol/l (SD 0.34).

**Conclusions:**

CSF protein and glucose levels are not affected by a systemic inflammatory response syndrome if there is no meningitis.

**Electronic supplementary material:**

The online version of this article (10.1186/s12987-018-0095-4) contains supplementary material, which is available to authorized users.

## Background

Meningitis is a substantial cause of morbidity and mortality in neonates. Abnormal cerebrospinal fluid (CSF) parameters are often used to predict meningitis and determine length and type of antibiotic therapy in neonates with a positive blood culture and negative CSF culture [1]. Currently, a positive CSF culture remains the golden standard for the diagnosis of neonatal bacterial meningitis in clinical practice. However, it may become negative within hours of antibiotic administration. Additionally, CSF culture has been shown to be of poor sensitivity for the diagnosis of bacterial meningitis. Therefore, clinicians must also rely on CSF glucose, white blood cell (WBC) count and protein concentration to determine the presence of meningitis [1, 2]. Paediatric cases of bacterial meningitis without initial CSF pleocytosis have been reported [3]. It has been estimated that paediatric meningitis without elevated CSF white cell count (pleocytosis) accounts for 0.5–12% of all cases of bacterial meningitis [3]. High CSF protein concentration may prognosticate for a poor outcome in neonates with bacterial meningitis [4].

High CSF protein and low CSF glucose have been associated with increased risk of sensorineural hearing loss in bacterial meningitis [5, 6]. Break down of the blood brain–barrier (BBB) in severe systemic inflammatory responses (SIR) without meningitis has previously been described but its relationship to brain damage has not been investigated [7]. Increased interleukin-1 levels in the CSF of neonates with sepsis without meningitis have previously been associated with white matter damage on magnetic resonance imaging [8]. Such inflammation-associated white matter damage manifested itself in water diffusion restriction with a reduction in the apparent diffusion coefficient (ADC) on diffusion-weighted imaging. The ADC gradually increased with increase in oedema, tissue rarefaction, decreased cell density and increase in extracellular space [9]. Such abnormalities appeared to predict later development of periventricular leukomalacia. C-reactive protein, which is released by hepatocytes in response to IL-6, which in turn is released in response to IL-1, has previously been associated with increased BBB permeability in conditions with inflammation of the meninges [10].

The aims of his study were to establish baseline data to enable an audit into appropriate management of abnormal CSF biochemistry measurements consistent with meningitis. The knowledge whether sepsis in itself is associated with abnormal CSF parameters without the presence of meningitis would enable those, who had meningitis and in whom appropriate action should be taken (full course of antibiotics, neurodevelopmental follow up), to be distinguished. Aims were also to establish whether CSF protein and glucose in sepsis without elevated CSF cell count and with negative CSF culture and/or PCR is different from CSF in patients without a systemic inflammatory response and different from the normal values of CSF biochemistry as reported in the literature (reference range) for neonates and to establish whether elevated CSF protein and reduced CSF glucose levels were associated with neurological damage. Some of the data presented here has previously been presented as a poster at the 9th Excellence in Pediatrics conference in Vienna in 2017 [11].

## Methods

### Patient population

All newborn infants (< 28 days of age) who had cerebrospinal fluid analysis recorded in the microbiology database without evidence of meningitis (CSF cell count < 20) over a 2-year period at Luton & Dunstable University Hospital, were included in the study. Patients were excluded from further analysis if bacteria, fungi or viruses were detected in the CSF by culture or PCR or if CSF biochemistry had not been analysed. Patients were divided into those who had a systemic inflammatory response at the time of admission: body temperature > 38.0 °C and/or C-reactive protein > 5 mg/dl (normal up to 5 mg/dl) or at any point in time after admission to the neonatal unit or paediatric wards, and those who did not. Organisms isolated in blood or urine culture were recorded. CSF parameters measured in the department for Clinical Biochemistry at the Luton & Dunstable University Hospital NHS Foundation Trust included total protein measured by a turbidimetric method [12]: the samples were preincubated in an alkaline solution containing EDTA, which denatures the protein and eliminates interference from magnesium ions. Benzethonium chloride is then added, producing turbidity. Glucose levels were measured by an enzymatic reference method with hexokinase [13]. Hexokinase catalyzes the phosphorylation of glucose to glucose-6-phosphate by ATP. Glucose-6-phosphate dehydrogenase oxidizes glucose-6-phosphate in the presence of NADP to gluconate-6-phosphate. No other carbohydrate is oxidized. The rate of NADPH formation during the reaction is directly proportional to the glucose concentration and measured photometrically. CSF white and red blood cell counts were determined in the Microbiology Department. The red blood cell count was included in our data collection and analysis to investigate the influence of potential contamination of the CSF by blood. CSF was cultured in all patients and standard PCR methodology was used to analyse CSF specimens for bacterial and viral pathogens where requested. We collected information on abnormal neurological features from follow up from inpatient records and letters generated following an out-patient appointment.

### Statistical methods

Categorical data (gender, presence of seizures and adverse neurological outcome, numbers of patients with organisms isolated from urine or blood cultures) were analysed using Chi square with Yates correction or Fisher’s exact test as appropriate. Continuous data with a parametric distribution (Gestational age, CSF protein and CSF glucose) were analysed using Student’s t-test for independent samples and continuous data with features of a non-parametric distribution (CSF white and red cell count) were analysed using Mann–Whitney testing. P-values were 2-tailed and for equal variances not assumed. The statistical software packages used were IBM SPSS version 20.0 and Epi-Info version 7.0 (CDC Atlanta, USA). A p-value of < 0.05 was taken as indicating a statistically significant difference.

## Results

A total of 244 newborn infants on the neonatal unit or the paediatric wards, who had lumbar punctures with no evidence of meningitis, were included in the study. Evidence of SIR was detected in 218 in that they had pyrexia and/or an elevated CRP at the time of admission. There was no evidence of SIR in 26 patients. A total of 16 patients (6.5%) had pathogens isolated from blood cultures and a total of 10 (4.1%) from urine cultures with no difference between groups with and without SIR. Clinical characteristics of included patients are listed in Table 1.

**Table 1:** Characteristics of newborn patients with and without a systemic inflammatory response (pyrexia on or after admission) who had a cerebrospinal fluid analysis and were without evidence of meningitis (there were no statistically significant differences (p > 0.05) between parameters in column two and three)

	No systemic inflammatory response (n = 26)	Systemic inflammatory response (n = 218)
Gestational age at cerebrospinal fluid analysis (weeks, mean, standard deviation)	38.7 (3.1)	39 (2.3)
Gender (male)	14	113
Number of patients with organisms isolated from blood	2^a^	14^b^
Number of patients with positive urine culture	2	8
Seizures	2	9
Adverse neurological outcome	1^c^	7^d^

^a^*E. coli* (1), group B streptococcus (1)

^b^Group B streptococcus (5). *E. coli* (4), *Staphylococcus aureus* (2) and Listeria (1), *Haemophilus influenzae* (1), *Klebsiella pneumoniae* (1)

^c^Mild gross motor delay

^d^Hypoxic ischemic encephalopathy (1), global developmental delay (4), neurogenic arthrogryposis (1), hypotonia (1)

CSF parameters were compared in patients with and without a SIR (see Table 2). All patients had CSF protein levels determined but 6 patients with an SIR (2.7%) had no CSF glucose determined. This percentage of unknown CSF glucose levels was not significantly different from the complete data collection in patients without SIR. Neurodevelopmental outcome was normal in most patients but was unknown in 16 patients with SIR and one patient without SIR. MRI brain imaging was only performed in 10 patients with SIR and one without SIR on the basis of presence of risk factors for brain injury. Three patients with SIR and one patient without had features consistent with hypoxic ischemic encephalopathy on cerebral MRI.

**Table 2:** Results of the analysis of cerebrospinal fluid (CSF) in patients with and without evidence of a systemic inflammatory response

	No systemic inflammatory response (n = 26)	Systemic inflammatory response (n = 218)	P-value for statistical significance of difference
CSF white cell count (cells/μl, median, range)	1 (0–20)	2 (0–18)	n.s.^a^
CSF red cell count (cells/μl, median, range)	51 (0–25,200)	216 (0–395,500)	n.s.
CSF protein level (g/l, mean, standard deviation)	0.89 (0.37)	0.92 (0.40)	n.s.
CSF glucose level (mmol/l, mean, standard deviation)	2.5 (0.34)	2.7 (0.83)^b^	n.s.

^a^*n.s.* not significant: p-value > 0.05

^b^In 6 patients there was no CSF glucose level available

## Discussion

In this retrospective audit we did not find a difference in CSF protein or glucose levels between patients with and without a systemic inflammatory response. This confirms results obtained in adults comparing patients with and without elevated CSF white cell count where the CSF/blood albumin ratio was not found to be elevated in patients with a SIR without elevated CSF white cell count [10]. Comparison of those parameters with data from healthy controls used to establish gestational age-specific reference ranges from the published literature, revealed that the levels we obtained were within the range of healthy newborns of a similar gestational age [14, 15]. There is good evidence that the systemic inflammatory response seen in sepsis can cause encephalopathy without meningitis being present [7]. The fact that the CSF protein was not elevated and CSF glucose levels were not reduced in our patients may have been due to a relatively mild systemic inflammation: none of our patients had multi-organ failure or died of sepsis. A specific elevation of cytokines in CSF as described in systemic inflammation without meningitis previously [8] may not have been accompanied by a disruption of the BBB but selective receptor-mediated transcytosis of specific cytokines like TNF alpha across the BBB or LPS diffusing through cell BBB cell layers and causing interleukin-1 production from astrocytes in the CNS without causing an elevation of total CSF protein [7]. The brain is protected by a BBB consisting of multiple layers of cell membranes, basement membranes and tight junctions to avoid interference with neuronal signals by pro-inflammatory cytokines and other plasma proteins which are able to change membranous ion transport and hence interfere with nerve signals [16]. Cytokines and nitric oxide metabolites generated in response to cytokines could also interfere with mitochondrial function in the brain thus causing increased glucose consumption through anaerobic glycolysis causing low CSF glucose with subsequent reduced brain function.

One could argue that the group without a systemic inflammatory response was too small to detect a statistically-significant difference. Because our study is to the best of our knowledge the first investigating this topic in this way a sample size calculation was not possible and the number of patients included in each group was the number of eligible patients identified during the study period to avoid any selection bias. Based on our data a sample size calculation based on equivalence showed that to exclude a statistically significant difference at the 5% significance level between groups one would have to include 550 patients (275 in each group) to be 80% certain that the limits of a two-sided 90% confidence interval will exclude a difference in means of CSF protein levels of more than 0.1 g/l (https://sealedenvelope.com/power/continuous-equivalence/accessed06/11/2017).

Even without an inflammatory response causing elevated CSF protein levels, systemic sepsis could cause white matter damage in the brain without meningitis. This can be attributed to lipopolysaccharides crossing the BBB and able to cause white matter injury through activation of microglial toll like receptor 4 [17]. The data obtained can reassure the clinician that the reference ranges for CSF protein and glucose levels for newborns as stated in the literature are likely applicable to newborns with features of a systemic inflammatory response as in early sepsis even if there is no meningitis. Future studies need to investigate whether an increase in CSF protein and reduction of CSF glucose is indicative of meningitis or encephalitis even in the absence of an elevated CSF white cell count, now that we know that in mere sepsis without elevation of CSF cell count these parameters are not affected by the systemic inflammatory response. On this basis an audit of appropriate management of abnormal CSF protein and glucose can proceed with disregard for the presence of sepsis.

## Conclusions

In newborn infants, CSF protein and glucose levels are not affected by a systemic inflammatory response syndrome if there is no concurrent meningitis.

## Additional file

**Additional file 1.** Raw data for the newborns included in the report.
